# Surface Plasmon Resonance (SPR) for the Evaluation of Shear-Force-Dependent Bacterial Adhesion

**DOI:** 10.3390/bios5020276

**Published:** 2015-05-26

**Authors:** Oleksandr Zagorodko, Julie Bouckaert, Tetiana Dumych, Rostyslav Bilyy, Iban Larroulet, Aritz Yanguas Serrano, Dimitri Alvarez Dorta, Sebastien G. Gouin, Stefan-Ovidiu Dima, Florin Oancea, Rabah Boukherroub, Sabine Szunerits

**Affiliations:** 1 Institute of Electronics, Microelectronics and Nanotechnology (IEMN), UMR-CNRS 8520, Université Lille 1, Cité Scientifique, 59655 Villeneuve d’Ascq, France; E-Mails: morjakzzz@gmail.com (O.Z.); phd.ovidiu.dima@gmail.com (S.-O.D.); rabah.boukherroub@univ-lille1.fr (R.B.); 2 Unité de Glycobiologie Structurale et Fonctionnelle (UGSF), UMR8576 du CNRS, Université Lille 1, 59655 Villeneuve d’Ascq, France; E-Mail: t.shkandina@gmail.com; 3 Institute of Cell Biology, National Academy of Sciences of Ukraine, 79005 Lviv, Ukraine; E-Mail: r.bilyy@gmail.com; 4 SENSIA SL, Poligono Aranguren, 9, Apdo. Correos 171, 20180 Oiartzun, Gipuzkoa, Spain; E-Mails: ilarroulet@seimcc.com (I.L.); aritz_yanguas@hotmail.com (A.Y.S.); 5 LUNAM Université, CEISAM, UMR 6230 du CNRS, 2, rue de la Houssinière, BP 92208, 44322 Nantes Cedex 3, France; E-Mails: dimitri.alvarez-dorta@univ-nantes.fr (D.A.D.); Sebastien.Gouin@univ-nantes.fr (S.G.G.); 6 Faculty of Applied Chemistry and Materials Science, University Politehnica of Bucharest, 1-7 Gheorghe Polizu, 011061 Bucharest, Romania; 7 National R&D Institute for Chemistry and Petrochemistry ICECHIM, 202 Splaiul Independentei, 060021 Bucharest, Romania; E-Mail: florino@ping.ro

**Keywords:** surface plasmon resonance (SPR), shear force enhancement, flow rate, *Escherichia coli* (*E. coli*), carbohydrates

## Abstract

The colonization of *Escherichia coli* (*E. coli*) to host cell surfaces is known to be a glycan-specific process that can be modulated by shear stress. In this work we investigate whether flow rate changes in microchannels integrated on surface plasmon resonance (SPR) surfaces would allow for investigating such processes in an easy and high-throughput manner. We demonstrate that adhesion of uropathogenic *E. coli* UTI89 on heptyl α-d-mannopyranoside-modified gold SPR substrates is minimal under almost static conditions (flow rates of 10 µL·min^−1^), and reaches a maximum at flow rates of 30 µL·min^−1^ (≈30 mPa). This concept is applicable to the investigation of any ligand-pathogen interactions, offering a robust, easy, and fast method for screening adhesion characteristics of pathogens to ligand-modified interfaces.

## 1. Introduction

Formation of biocomplexes (e.g., antigen–antibody, DNA–DNA, proteins–nucleic acids, *etc.*) is an affinity reaction present in most important biochemical processes involved in nature. This principle has found widespread use in designing bioanalytical assays. One of the widely used bioanalytical techniques for real-time monitoring of affinity interactions between immobilized biological components on a transducer surface and target molecules in solution is Surface Plasmon Resonance Spectroscopy (SPR) [1,2]. Over the years, SPR has shown its potential as an alternative to conventional biochemical methods for studying a wide range of bimolecular interactions with high sensitivity and selectivity [3,4,5]. SPR sensors have also been demonstrated to allow quantification of a wide range of pathogens through the detection of formed protein toxins [6,7,8], small molecule toxins [9,10], or the whole bacteria via surface-immobilized specific antibodies or bacteriophages [11,12,13,14,15]. Next to antibodies and bacteriophages, carbohydrate derivatives have been considered as possible SPR targets for bacterial toxins and pathogens [8,16,17]. Glycan-modified SPR interfaces were used by Bouckaert *et al.* for the determination of the affinity of FimH-carbohydrate interactions [18]. Szunerits and Bouckaert showed recently that the adhesion of uropathogenic and enterotoxigenic *Escherichia coli* (*E. coli*) clinical isolates, both of which express structurally characterized fimbrial adhesions, can be studied conveniently on glycan-modified SPR interfaces [17].

In this paper, we ask whether SPR, in addition to its confirmed quantitative character, has potential as an analysis tool for investigating the impact of shear on the adhesion of pathogens to surfaces and allows to extract information about the adhesion dynamics of pathogens. Bacterial cells are often dispersed in a flowing carrier fluid (e.g., water, urine, or blood); thus studying the dynamics of bacterial adhesion under controlled conditions of both flow and chemical environment is particularly relevant. 

The impact of shear caused by the flow of fluids on bacterial adhesion to biotic and abiotic surfaces has indeed been considered in the past [19,20,21,22]. Thomas and co-workers observed that the adhesion of *E. coli* to mannose-coated surfaces was enhanced by shear stress and attributed this phenomenon to the formation of force-enhanced allosteric catch-bonds between the type-1 fimbrial adhesion and surface-attached mannose moieties [20,22,23]. Experimental evidence for the shear-force-dependent adhesion was provided from data using molecular force spectroscopy with purified FimH and demonstrated that a tensile force extends the lifetime of the bond established between FimH and the mannose receptor [24]. More recently, Klinth *et al.* showed that the adhesive properties of pilated *E. coli* and specifically their dependence on pH can be investigated by force measurements with optical tweezers and SPR using purified P pili as well as whole bacteria [25].

In this paper, the dynamics of adhesion of *E. coli* onto heptyl α-D-mannopyranoside (HM)-modified gold SPR interfaces is investigated. We focus on the effect of shear force on the initial adhesion of *E. coli* UTI89 mediated by FimH, a mannose-recognizing adhesin found at the tip of the type 1 pilus [26,27]. The data indicate that the presence of HM has a strong bearing on the measured adhesive capacity of *E. coli* UTI89. To examine the specificity of the bacterial adhesion for the sugar-modified plasmonic interfaces, we also interacted a type-1 fimbriated *E. coli* strain, UTI89 Q113K, carrying a mutation in FimH (glutamine 133 to lysine) and rendering it dysfunctional for mannose-binding, as well as UTI89 ∆*fimH*, with a deletion of the *fimH* gene from the *fim* operon, which therefore is hampered in the initiation of type-1 fimbrial biogenesis [17,28,41].

## 2. Experimental Section

### 2.1. Materials

11-mercaptoundecanoic acid (MUA), *N*-hydroxysuccinimide (NHS), *N*,*N'*-dicyclohexylcarbodiimide (DCC), dimethyl formamide (DMF) and ethanol were purchased from Aldrich and used as received. Aminoheptyl α-D-mannopyranoside (HM) was synthesized as reported previously [29].

### 2.2. Formation of Glycans-Modified SPR Interfaces

Gold-based SPR interfaces were provided by Sensia and used as received. Carboxylic acid-terminated self-assembled monolayers were formed through immersion of the gold substrate in an ethanolic solution of 11-mercaptoundecanoic acid (1 mM) for 24 h. The samples were rinsed with ethanol (three times) and water (three times) and dried under argon. 

The carboxylic acid-terminated surface (Au-COOH) was immersed in DMF solution (20 mL) of DCC (2 mM) and NHS (2 mM) for 40 min to activate the COOH groups. The modified interfaces were further reacted overnight in a solution of aminoheptyl α-D-mannopyranoside (2 mM) in DMF under stirring to form the amide linkage. The interface was rinsed twice with DMF and then ethanol and thereafter dried under a gentle stream of nitrogen.

### 2.3. Determination of the Amount of Glycans on the SPR Interfaces

The amount of glycan on gold was determined by treatment with a phenol/H_2_SO_4_ solution as described previously [30]. First, a calibration curve for aminoheptyl α-D-mannopyranoside in solution was established using a phenolic aqueous solution (5 wt %, 60 µL), concentrated H_2_SO_4_ (900 µL) which was added to an aqueous glycan solution (60 µL), stirred for 10 min, and then an absorption spectrum was recorded against blank sample without glycan. The absorbance of the solution was measured at two wavelengths, λ_1_ = 485 and λ_2_ = 570 nm, and the absorbance difference (A_485_–A_570_) was plotted as a function of the concentration of the glycan. The glycan-modified SPR interface was treated with phenol/H_2_SO_4_ following the same protocol described above.

### 2.4. Instrumentation 

#### 2.4.1. SPR

SPR measurements were performed with a commercial available SPR instrument called “Indicator” (provided by SENSIA, Oiartzun, Spain), working at a wavelength of 650 nm. The instrument is equipped with a two-channel flow cell system that can be in a first approach modeled as a 12 mm long cell with an inner diameter of 0.5 mm. The flow speed can be adjusted from 4 to 100 µL·min^−1^. Each of the loops has a volume of 60 µL. The prisms used have a refractive index of *n* = 1.569 (HBAK1, Schott Global) and have been modified by a Ti adhesion layer of 2 ± 0.5 nm and a gold thin film of 47 ± 2 nm, both deposited by sputtering under vacuum.

#### 2.4.2. UV/Vis Measurements

Absorption spectra were recorded using a spectrophotometer (Perkin Elmer Lambda UV/Vis 950) in plastic cuvettes with an optical path of 10 mm. The wavelength range was 400–800 nm.

#### 2.4.3. X-Ray Photoelectron Spectroscopy

X-ray photoelectron spectroscopy (XPS) experiments were performed in a PHl 5000 VersaProbe—Scanning ESCA Microprobe (ULVAC-PHI) instrument at a base pressure below 5 × 10^−9^ mbar. Monochromatic AlK_α_ radiation was used and the X-ray beam, focused to a diameter of 100 µm, was scanned on a 250 µm × 250 µm surface, at an operating power of 25 W (15 kV). Photoelectron survey spectra were acquired using a hemispherical analyzer at pass energy of 117.4 eV with a 0.4 eV energy step. Core-level spectra were acquired at pass energy of 23.5 eV with a 0.1 eV energy step. All spectra were acquired at 90° between X-ray source and analyzer and with the use of low-energy electrons and low-energy argon ions for charge neutralization. After subtraction of the Shirley-type background, the core-level spectra were decomposed into their components with mixed Gaussian–Lorentzian (30:70) shape lines using the CasaXPS software. Quantification calculations were performed using sensitivity factors supplied by PHI.

### 2.5. Bacteria

The wild-type UTI89 *E. coli* strain carries a few hundreds of mannose-sensitive type-1 fimbrial adhesins FimH. A strain called UTI89 Q133K had been made dysfunctional for mannose binding through a glutamine 133 to lysine mutation [17,28]. UTI89 ∆f*imH* bacteria carry no fimbriae. Upon plating on LB-agar complemented with the appropriate antibiotics, the bacteria were inoculated in LB plus antibiotics for 48 h static growth at 37 °C, to optimize fimbrial expression. The bacterial pellet was obtained upon centrifugation, washed three times in phosphate-buffered saline (PBS), and then solubilized to an optical density at 600 nm (OD_600nm_) of about 10 (agreeing with about 1 × 10^10^ colony forming units/mL) for spectrometric determination of the optical densities. The bacteria were further diluted to 1 × 10^8^ cfu/mL using PBS for SPR studies.

## 3. Results and Discussion

### 3.1. Modification of Gold SPR Interfaces with Aminoheptyl α-D-Mannopyranoside (HM)

The interaction of *E. coli* UTI89 with host tissue is known to be mediated by the 30 kDa mannose-specific FimH protein, which is located on the tip of type 1 fimbriae [31]. Previous results demonstrated that the low micromolar affinity of *E coli* UTI89 to simple mannose can be overcome by using synthetic monovalent mannosides bearing hydrophobic [32] or simple alkyl [26] aglycons in the anomeric position. Aminoheptyl α-D-mannopyranoside (HM) was in this respect identified as a particular promising ligand [29] and used in this work for the modification of SPR interfaces. 

**Figure 1:**
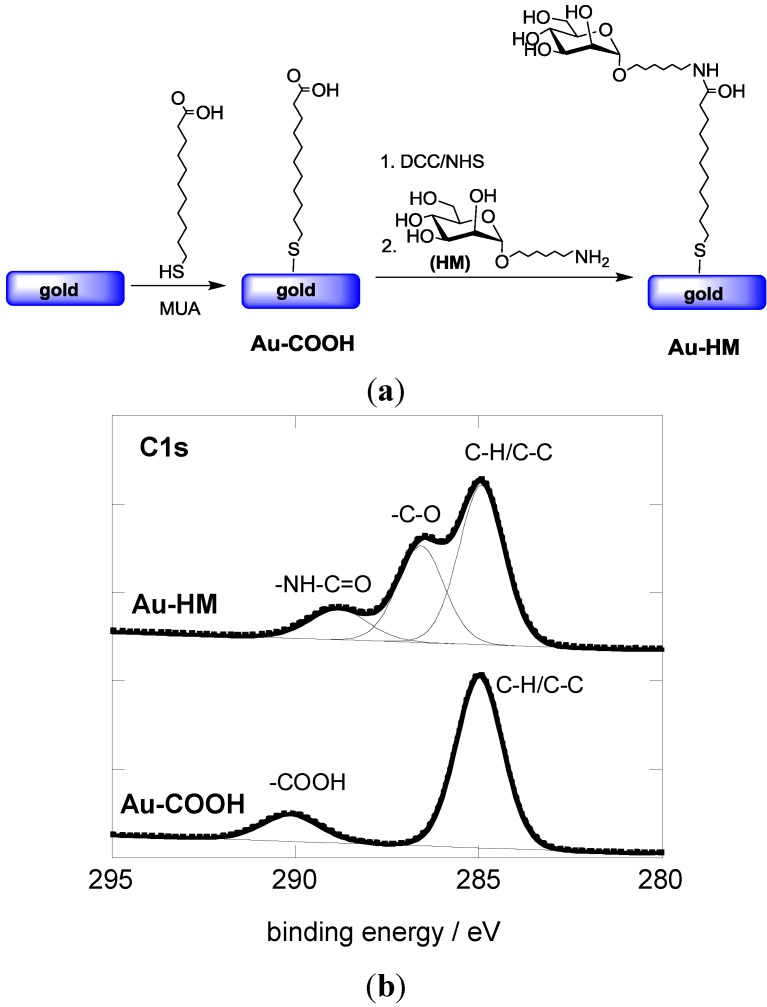
(**a**) Reaction scheme for the formation of aminoheptyl α-D-mannopyranoside modified SPR interfaces; (**b**) C1s core level XPS spectra of Au-COOH and Au-HM interfaces.

The covalent linking of HM to gold-based SPR interfaces was achieved in a two-step procedure (Figure 1a). First, an acid-terminated self-assembled monolayer (Au-COOH) was formed through immersion of the gold interface in an ethanolic solution of 11-mercaptoundecanoic acid (MUA) for 24 h. Figure 1B shows the C1s core level photoemission spectrum for the resulting Au-COOH interface. The presence of the aliphatic carbon atoms results in a peak at 285.0 eV, while the feature at 289.1 eV is due to the terminal carboxylic groups.

Aminoheptyl α-D-mannopyranoside was successfully coupled to the Au-COOH interface using peptide linkage chemistry between the terminal -NH_2_ groups of HM and NHS-activated ester groups of the Au-COOH interface (Figure 1a). The C1_S_ core level spectrum of the Au-HM interface shows next to the peak at 285.0 eV (aliphatic carbon atoms) contributions at 286.2 eV and 288.1 eV, characteristic of C–O bands of the integrated glycans and the formed amide bond, respectively (Figure 1b). The degree of sugar loading was determined using the classical phenol-sulfuric acid method as described in several papers by us [30,33]. A loading of (8.5 ± 0.6) × 10^14^ molecules·cm^−2^ was thus determined, in accordance with other reports on mannose-modified SPR surfaces [33,34].

### 3.2. Adhesion Behavior of E. coli UTI89 to Au-HM under Different Flow Rates

**Figure 2:**
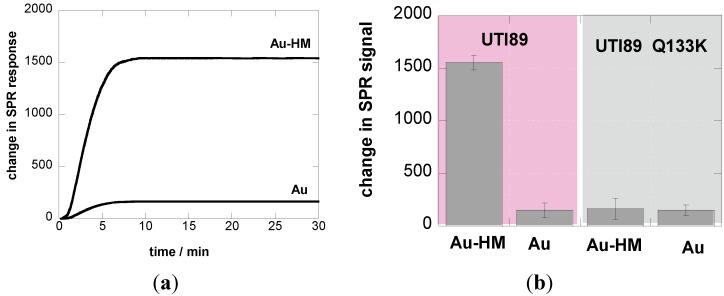
(**a**) SPR sensogram of affinity interaction of *E. coli* UTI (10^8^ cfu/mL) with Au and Au-HM interfaces; flow rate = 20 µL/min; (**b**) bar graph diagrams of the change in SPR signals of Au and Au-HM interfaces upon interaction for 30 min at a flow rate = 20 µL/min with UTI 89 and UTI89 Q133K.

The interaction between *E. coli* (10^8^ cfu/mL) and Au-HM interfaces was investigated in real time using label-free interaction analysis in an SPR assay format (Figure 2). Figure 2a shows the change in the SPR signal over 30 min on Au and Au-HM interfaces upon interaction with *E. coli* UTI89. The binding ability of *E. coli* UTI89 to Au-HM was significantly larger than on gold alone, in line with the well-known glycans-mediated increase in pathogen adhesion. To underline the specific aminoheptyl α-D-mannopyranoside-*E. coli* UTI89 interaction, the adhesion behavior of a strain called UTI89 Q133K, made dysfunctional for mannose binding through a glutamine 133 to lysine mutation [17,28], was investigated in addition. As seen in Figure 2b, UTI89 Q133K shows no sugar specific interaction and the change in the SPR signal recorded is the same for Au and Au-HM interfaces.

The experiments carried out so far were performed at a flow rate of 20 µL·min^−1^ in line with other SPR experiments [35]. Fluid flow is, however, an important factor in microbial deposition [36,37] and can be controlled in the flow chamber of the SPR cell by changing the flow rate. An increase in flow rate is expected to stimulate attachment of pathogens due to an increase in microbial transport towards the substrate. In other words, an increase in flow rate results in faster adhesion kinetics, where the SPR signal is saturating more rapidly. The increased shear force might, in addition, lead to a larger amount of bacteria adhering to the SPR interface, observable as an increased height of the plateau in the sensogram. There is, however, a critical flow rate beyond which detachment rather than adhesion of bacteria will occur. This critical flow rate varies from strain to strain and depends in addition on the involved surface. In the case of *E. coli*, the shear-force properties of FimH have been demonstrated previously using yeast mannan, mannose linked to bovine-serum-albumin, or guinea pig red blood cells, all of which are receptors for type 1 fimbriae [22,24,38,39].

**Figure 3:**
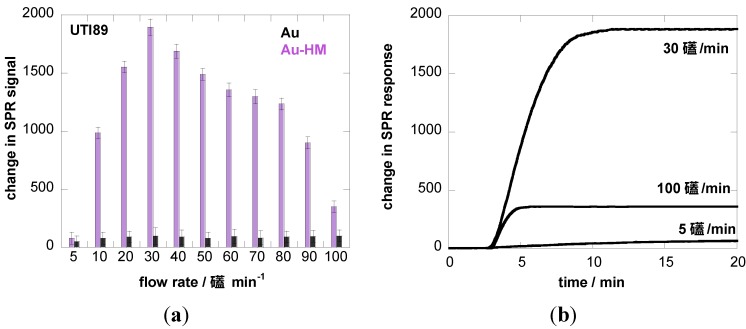

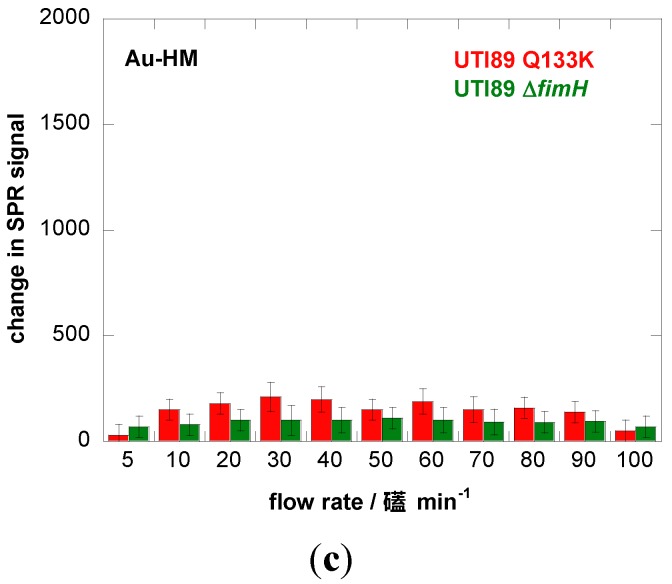
Binding affinity of *E. coli* UTI89 (10^8^ cfu/mL) to Au and Au-HM as a function of flow rate. (**a**) Bar graph of change in SPR signal upon addition of *E. coli* UTI (10^8^ cfu/mL); (**b**) SPR sensogram for three different flow rates; (**c**) binding affinity of *E. coli* UTI89 Q133K (10^8^ cfu/mL) and UTI89 ∆*fimH* (10^8^ cfu/ml) to Au-HM surfaces as a function of flow rate.

We therefore investigated the influence of flow rate on the binding strength of *E. coli* UTI89 onto Au and Au-HM. The microfluidic channel used in combination with the SPR setup allowed us to vary the flow rate between 5 and 100 µL·min^−1^, which corresponds to a shear of ≈5–100 mPa (0.005–0.1 pN/μm^2^), a range used by others [22,40]. Figure 3a shows the binding *of E. coli* UTI89 at different flow rates to Au and Au-HM. In the case of unmodified gold SPR interfaces, the flow rate has no effect on the change in the SPR signal and thus the adhesion strength of *E. coli* UTI89. In fact, almost no adhesion is observed for gold only. This was in contrast to the Au-HM interface, where *E. coli* adhesion increased strongly with increasing flow rate from 5–30 µL·min^−1^, where a maximum in SPR response was reached. Higher flow rates resulted in a gradual decrease in the SPR signal. At 90 µL·min^−1^, about half of the maximal value was reached, and completely dropped to very low values at 100 µL·min^−1^. Interestingly, the increase in adhesion until a flow of 30 µL·min^−1^ is more rapid than the decrease in adhesion above the maximum value. The results presented in Figure 3a clearly indicate the mannose-specific mechanism of the flow-rate-dependent *E. coli* adhesion to Au-HM. From Figure 3b, the time to reach a maximal adhesion is ≈5–8 min and depends on the flow rate. While the protein FimH is highly conserved in *E. coli* UTI89, mutations in the binding pocket could, however, influence adhesion under shear flow. We were thus intrigued to investigate the binding characteristics of strain UTI89 Q133K and of UTI89 ∆*fimH*. In the case of bacterial strain UTI89 ∆*fimH*, the capacity to bind to bladder cells is lost due to the incapacity to form normal type-1 pili in the absence of the FimH protein [41]. As seen in Figure 3c, no shear-force enhanced adhesion could be observed for this strain: while there is a small increase in the SPR signal at a flow rate of 30 µL·min^−1^ in the case of UTI89 Q133K, the SPR signal upon the addition of UTI89 ∆*fimH* is completely constant for the different flow rates measured. This is line with reports that fimbrial proteins and in particular the FimH adhesin play a key role in the adherence of uropathogenic *E. coli* to urothelial surfaces. A loss of fimbriae results in the impossibility of adhering to mannose-carrying surfaces and ultimately to the formation of biofilms.

## 4. Conclusions

In conclusion, the use of microchannels in SPR instrumentation has been shown to be adequate to investigate the shear-force- and surface-ligand-based microbial attachment capacities of *E. coli* UTI89 strains. Changing the flow rate between 5 and 100 µL·min^−1^ (shears of 5–100 mPa) shows that the strongest affinity of *E. coli* UTI89 to aminoheptyl α-D-mannopyranoside modified surfaces occurs at 30 µL·min^−1^. The results presented in this work clearly point towards the influence of flow rate on the binding capability of pathogens. This study also indicates that flow-rate-induced adhesion requires specific ligand-receptor interaction, as between surface-anchored mannose units and FimH present at the distal end of *E. coli* UTI89. Mutation at this very end, as for *E. coli* UTI89 Q133K, results in a predominant loss of adhesion and the hampering of type-1 fimbrial biogenesis, through *fimH* gene deletion, which completely abolishes shear-force-based adhesion. It is hoped that this work will widen the possibilities of studying bacterial colonization with the ultimate goal of adding to the understanding of the complex ways in which bacteria adhere to surfaces. 
